# Evidence of cortical thickness reduction and disconnection in high myopia

**DOI:** 10.1038/s41598-020-73415-3

**Published:** 2020-10-01

**Authors:** Ya-Jun Wu, Na Wu, Xin Huang, Jie Rao, Li Yan, Ling Shi, Hui Huang, Si-Yu Li, Fu-Qing Zhou, Xiao-Rong Wu

**Affiliations:** 1 Department of Ophthalmology, The First Affiliated Hospital of Nanchang University, Nanchang, 330006 Jiangxi China; 2 Department of Radiology, The First Affiliated Hospital of Nanchang University, Nanchang, 330006 Jiangxi China

**Keywords:** Retinal diseases, Eye abnormalities

## Abstract

High myopia (HM) is associated with impaired long-distance vision. accumulating evidences reported that abnormal visual experience leads to dysfunction in brain activity in HM even corrected. However, whether the long-term of abnormal visual experience lead to neuroanatomical changes remain unknown, the aim at this study is to investigate the alternation of cortical surface thickness in HM patients. 82 patients with HM (HM groups), 57 healthy controls (HC groups) were recruited. All participants underwent high-resolution T1 and resting-state functional magnetic resonance imaging (MRI) scans. The cortical thickness analysis was preformed to investigate the neuroanatomical changes in HM patients using computational anatomy toolbox (CAT 12) toolbox. Compare with HCs, HM patients showed decreased the cortical surface thickness in the left middle occipital gyrus (MOG), left inferior parietal lobule (IPL), right inferior temporal gyrus (ITG), right precuneus, right primary visual area 1 (V1), right superior temporal gyrus (STG), right superior parietal lobule (SPL), right occipital pole, and right the primary motor cortex (M1), and increased to the parietal operculum (OP4) (P < 0.01, FWE-corrected), the mean cortical thickness of right orbitofrontal cortex (OFC), right dorsolateral prefrontal cortex (DLPFC) and right subcallosal cortex showed negatively correlation between clinical variables (axis length (ALM), the average macular thickness (AMT), keratometer (KER) 1, KER2, the mean KER, the mean macular fovea thickness (MFK), the refractive diopter) in HM patients. Our result mainly provided an evidence of cortical thickness reduction and disconnection in visual center and visual processing area, and cortical thickness increase in left multimodal integration region in HM patients. This may provide important significance of the study of the neural mechanism of HM.

## Introduction

High myopic (HM) is commonly worldwide, especially in young Asians, ranges from 6.8 to 21.6%^1,2^. HM is often associated with pathological myopia (PM) or serious eye complications such as retinal detachment (RD), macular hole (MH)^3^, cause irreversible visual impairment, even blindness. Recent study has shown that the visual function of patients with HM is abnormal compared with emmetropia, even if the corrected visual acuity is the same, this study reported that lower functional connectivity (FC) in the posterior cingulate cortex/precuneus, and functional changes in many other brain regions in HM patients, they believe that abnormal visual experience can lead to abnormal brain structure and function^4^. Also, in our previous study, we observed that distributed alteration of amplitude of low-frequency fluctuation (ALFF)^5,6^ and decreased short- and long-range functional connectivity density (FCD)^4^ in HM patients.

Besides, previous voxel-based morphometry (VBM) studies have suggested that increased concentration of white matter (WM) primarily in the calcarine area, but normal grey-matter (GM) concentration in human with late-onset myopia^7^. In early blindness, significant increased cortical thickness, which they believe may be due to the degeneration of cortical synapses caused by abnormal visual experience in a long time^8^. HM patients have long been in the abnormal visual state of myopia, therefore, we speculate that the cortical thickness of HM patients may get changed. However, up to now, there has still been no study to explore the relationship between HM and the change of cortical thickness in the whole-brain grey.

In this study, we hypothesis that there had cortical thickness reduction in HM patients and it associated with abnormal visual experience and/or local susceptibility. In fact, cortical thickness measurement is regarded as a good candidate for detecting neuronal loss or degradation. Cortical thickness mapping calculated as the distance between the white matter-gray matter (WM-GM) surfaces (approximately 1.6–4.5 mm^9–11^), as a fully automated method based on the projection-based thickness (PBT) measurement^9^ to provide a local measure of GM within the cortex^12^. It was more sensitive when detecting the morphological changes in the region^13,14^. Therefore, we deeply believe in it is more specific to monitor the thickness of cerebral cortex in the methods of observing brain structure.

Hence, in this study, we investigate the whole-cortical surface thickness and related functional connectivity in MH patients, in order to provide important basis for searching for the alteration of cortical structural plasticity in the context of myopic related abnormal visual experience.

## Materials and methods

### Subjects

All subjects (n = 139) were sequentially recruited from August 2016 to July 2017 at the First Affiliated Hospital of Nanchang University in all, 82 HM individuals and 57 healthy control subjects (HCs).All participants excluded abnormal neurological diseases (cerebral hemorrhage, mental disorder, stroke and other diseases) through medical history collection, physical examination, etc. All participants had no evidence of T2-weighted imaging (T2WI) and fluid-attenuated inversion recovery (FLAIR) imaging. All participants were young adults that were used to wearing spectacles with no bad habit of wearing spectacles. All participants had received tests of the same environment. All patients had signed the informed consent form. All procedures were conducted in accordance with the guidelines on the Declaration of Helsinki, and ethical approval was approved by the medical ethics committee of the first affiliated hospital of Nanchang university in Jiangxi province, China.

The following inclusion criteria were applied: (1) Binocular refractive diopter less than − 6.00 and the corrected visual acuity (VA) greater than 1.0. (2) There are no other ophthalmologic conditions such as amblyopia, squint, cataract, glaucoma, retinal degeneration, etc. (3) The patients have not received any eye surgery, no long-term use of eye drop. (4) All patients agreed to MRI related examination and optical coherence tomography (OCT), an ultrasonometry and other ophthalmic examinations. The exclusion criteria for HM patients are as follows: (1) Monocular high myopia and super high myopia (refractive diopter less than − 10.00). (2) HM with amblyopia, strabismus, glaucoma, cataract, RD, MH and other eye diseases. (3) HM was accompanied by abnormal fundus changes, such as macular hemorrhage, subretinal neovascularization, leopard pattern fundus, Fuchs spot, etc. (4) HM patients have undergone ophthalmic surgery. (5) The patient's body part contains metal that cannot be removed and cannot be detected by MRI. (6) Patients who are difficult to cooperate with relevant ophthalmic examination.

HCs were demographically matched by sex, age and education level from respondents to flyers who were randomly recruited from the urban community of Nanchang city, all HCs had no ocular diseases with uncorrected or corrected VA greater than 1.0.

Ying Zhang et al. (our preliminary study)^15^ Used MRI technology in a study on cortical thickness of white matter hyper signal lesions in middle-aged and elderly people, in this study, we follow the methods of Zhang:

### MRI data acquisition

All MRI data were obtained using a 3-TeslaTrio MR imaging scanner system (Trio Tim, Siemens Medical Systems, Erlangen, Germany). These included conventional T2WI and FLAIR imaging for diagnostic and radiological assessment and high-resolution T1-weighted imaging (T1WI) for cortical surface complexity analyses. The following imaging parameters were used in the study: (1) three-dimensional high-resolution T1WI: repetition time/echo time = 1900/2.26 ms, field of view = 215 × 230 mm, matrix = 240 × 256, and 176 sagittal slices with a 1.0-mm thickness; (2) turbo spin echo-imaging sequence for T2WI scans: repetition time/echo time = 5100/117 ms, field of view = 240 × 240 mm, matrix = 416 × 416, number of excitations = 3, echo train length = 11, 22 axial slices with a 6.5-mm thickness; and (3) FLAIR imaging: repetition time/echo time/inversion time = 7000/79/2500 ms, 50 slices, 240 × 217 matrix, 0.43 × 0.43 × 2 mm^3^ voxels. The MRI data were visually inspected for obvious artifacts arising from subject motion and instrument malfunction^15^.

### Structural data processing and cortical surface extraction

High-resolution T1WI data was preprocessed using an automated program in the Computational Anatomy Toolbox (CAT12) to generate a cortical surface that provided cortical thickness measurement. This processing runs MATLAB 8.4 (R2014b; MathWorks, Natick, MA, USA). In short, T1WI data is the tissue of bias-field correction, skull dissection, alignment with the Montreal Neurological Institute standard space (MNI-152 template), segmentation into GM, WM, or cerebrospinal fluid within the same reproductive model^15,16^. In order to improve registration accuracy, a group-specific template was established using the Lie Algebra Diffeomorphic Anatomical Registration Through Exponentiated (DARTEL) algorithm in statistical parameter mapping (version12), and the non-linear transformation of individuals to the template was calculated^15^.

CAT12 is a new fully automatic method that measures cortical thickness and reconstructs the central surface in one step^9,15^. The program uses tissue segmentation to estimate the WM distance and uses the neighbor relationship described by the WM distance to project a local maximum (equal to the thickness of the cortex) to other GM voxels. This PBT allows the management of partial volume information, sulcal blurring, and sulcal asymmetries without the need for explicit groove reconstruction through bone or thinning methods. For inter-subject comparisons, the local complexity maps were re-parameterized to a common coordinate system and smoothed using a 15-mm Gaussian heat kernel^10,15,17^. For quality control, we excluded 7 left hemisphere and 6 right hemisphere data of HM subjects and three healthy controls based on the poor quality of their cortical surface reconstructions.

### Statistical analysis of cortical thickness and region of interest selection

We used the same method as in previous studies^15^: one-sample t-tests were performed for the spatial maps of global cortical thickness in HM and HCS groups. And then, Group differences in global cortical thickness were compared between the HM and HCS groups using a two-sample t-test. Then, regional differences were compared using a general linear model in the statistical analysis menu of CAT12, these results were used as indicators of changes related to HM. The threshold value P < 0.01 and a family-wise error (FWE) correction was applied.

The clusters of significant differences in the cortical thickness were considered to be the seeds for further FC analysis. We constructed a label based on the peak vertex for each of the selected clusters, then registered for the EPI template.

### Functional data processing and FC analysis

All the functional data processing were done with a toolbox for Data Processing & Analysis of Brain Imaging (DPABI, https://www.rfmri.org/DPABI). Main steps including discarded first 10 volumes, corrected slices time, realigned head motions, normalized to the Montreal Neurological Institute (MNI) standard space, resampled to 3 mm cubic voxels, and spatial smoothed. After excluded the data with motion exceeded 2 mm or 2° in the translation or the rotation, band-pass filter (0.01–0.1 Hz) and nuisance linear regression for reduction in motion effects (including white matter signal, cerebrospinal fluid signal and head motion parameters of the Friston 24 parameter model).

Seed-based FC analysis was conducted in DPABI based on the spherical ROI (peak coordinates from cortical thickness analysis, radius = 10 mm). FC maps were produced by computing the *Pearson* correlation coefficients between the seed region and the time series from all the other brain voxels and converted to z-values to improve normality.

### Statistical analysis of FC

Firstly, an one-sample t-test was using to show the significantly positively correlated regions. Group differences between the HM and HCs were evaluated with a general linear model analysis, within the mask of positive connectivity regions of seed (voxel level, P < 0.01; GRF corrected at the cluster level, P < 0.01). We focused only on positive connectivity in the FC analysis because it remains an unsettled debate whether the autocorrelation is an artifact of global signal regression.

### Statistical analysis of clinical measurements and correlation analysis

A two-sample t-test was used to compare the differences in age, education, and overall average thickness of Statistical Product and Service Solutions V13.0 (SPSS Inc., Chicago, IL, USA). In addition, a Chi-square test was used for comparing gender and dominant hand^15^.

Finally, we used a simple linear regression model to assess the correlation between CAT12 to evaluate the relationship between visual assessment of HM and cerebral cortical thickness in HM group. Age, gender and education level were used as meaningless covariance. The threshold value was set to the significance level of P < 0.01 and was compared several times by FWE-corrected^15^.

## Results

### Demographic and clinical measurements

#### Demographic and clinical measurements

For the HM group, the range of age was 24.37 ± 3.62, and for HC group was 22.29 ± 1.27; As for refractive diopter (D) (OD), which was − 6.41 ± 2.01, not applicable (NA) for HM and HC group, respectively; And for refractive diopter (D) (OS), which was − 6.23 ± 2.15, NA for HM and HC group, respectively. For the HM group, the ALM (mm) was 26.02 ± 1.09, 25.90 ± 1.19 for OD and OS, respectively; For the HC group, the ALM (mm) was 22.13 ± 2.01, 23.01 ± 1.06 for OD and OS, respectively; Furthermore, the mean HM KER was 43.57 ± 1.33 D and 43.53 ± 1.33 D for OD and OS, respectively; As for the mean HC KER was 43.01 ± 1.03 D and 42.98 ± 1.56 D for OD and OS, respectively; The MFK (µm) (OD) was 242.4 ± 33.68, NA for HM and HC group, respectively; The MFK (µm) (OS) was 251.00 ± 23.67, NA for HM and HC group, respectively; Moreover, for HM group, the AMT (µm) (OD) and OS was 263.53 ± 21.80, 266.74 ± 17.85, respectively; But for HC group, the AMT was unavailable, all the details were showed in Table 1.

**Table 1:** Demographic and clinical measurements.

Conditions	HM (mean ± SD)	HC (mean ± SD)
Male/female	30/52	27/30
Age (years)	24.37 ± 3.62	22.29 ± 1.27
Refractive diopter (D) (OD)	− 6.41 ± 2.01	NA
Refractive diopter (D) (OS)	− 6.23 ± 2.15	NA
ALM (mm) (OD)	26.02 ± 1.09	22.13 ± 2.01
ALM (mm) (OS)	25.90 ± 1.19	23.01 ± 1.06
mean KER (D) (OD)	43.57 ± 1.33	43.01 ± 1.03
mean KER (D) (OS)	43.53 ± 1.33	42.98 ± 1.56
MFK (µm) (OD)	242.4 ± 33.68	NA
MFK (µm) (OS)	251.00 ± 23.67	NA
AMT (µm) (OD)	263.53 ± 21.80	NA
AMT (µm) (OS)	266.74 ± 17.85	NA

*HM* high myopic control, *HC* healthy control, *D* diopter, *OD* oculus dexter, *OS* oculus sinister, *ALM* axis length, *KER* keratometer, *MFK* macular fovea thickness, *AMT* average macular thickness, *NA* not applicable.

### Cortical thickness reduction in HM group

**Figure 1:**
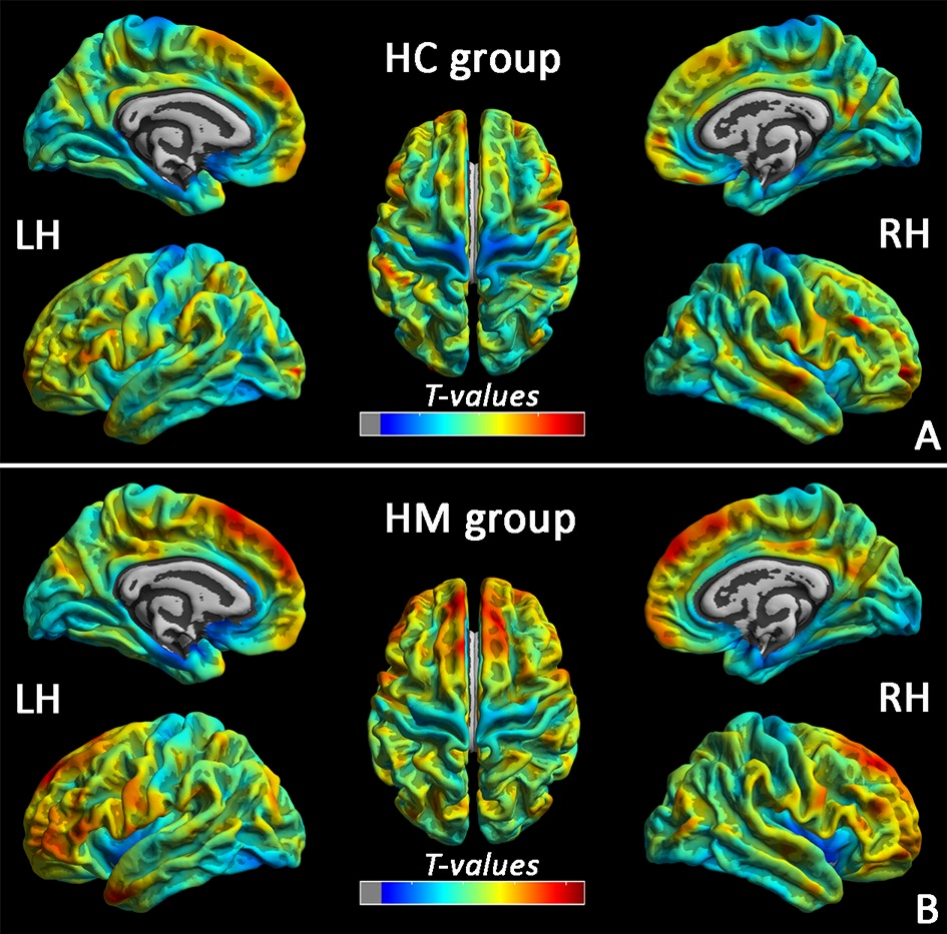
Cerebral cortical thickness patterns in with HC (**A**) and HM (**B**) subjects (*LH* light hemisphere, *RH* right hemisphere, *HM* high myopic control, *HC* healthy control).

**Figure 2:**
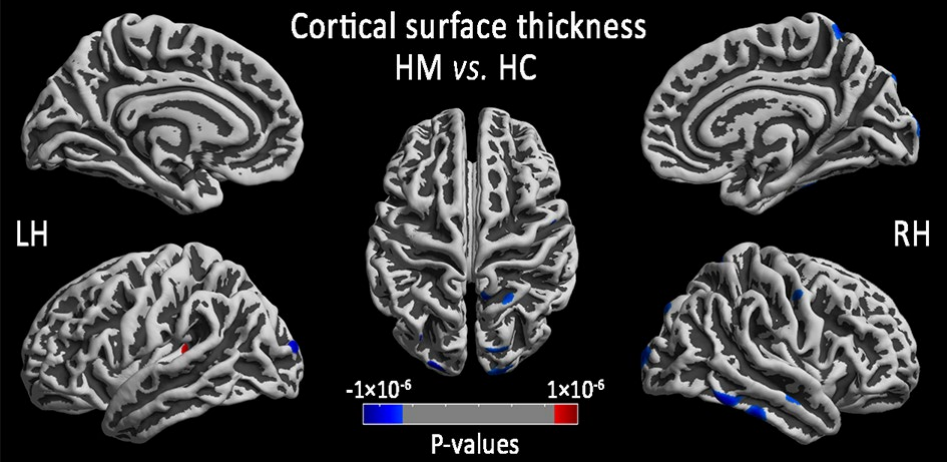
Comparison of local cortical thickness between the HM group and the HC group (P < 0.01, FWE-corrected). Prussian blue indicates a significantly lower cortical thickness value, and crimson indicates a significantly higher cortical thickness value (*LH* light hemisphere, *RH* right hemisphere, *HM* high myopic control, *HC* healthy control).

The patterns of mean cerebral cortical thickness for the HC group (Fig. 1A) and the HM group (Fig. 1B). Compare with HCs, HM patients showed decreased the cortical surface thickness in the left middle occipital gyrus (MOG), left inferior parietal lobule (IPL), right inferior temporal gyrus (ITG), right precuneus, right primary visual area 1 (V1), right superior temporal gyrus (STG), right superior parietal lobule (SPL), right occipital pole, and right the primary motor cortex (M1), and increased to the parietal operculum (OP4) (P < 0.01, FWE-corrected) (Fig. 2 and Table 2).

**Table 2:** Comparison of regional cortical thickness between the HM and HC.

Location	Cluster size	Peak MNI			T-values
		X (mm)	Y (mm)	Z (mm)	
**Left hemisphere: HM (n = 79) vs. HC(n = 57)**					
Middle occipital gyrus (MOG)	148	− 7	− 100	− 5	− 4.95
Inferior parietal lobule (IPL)	29	− 21	− 91	25	− 4.23
Parietal operculum (OP4)	92	− 36	− 39	11	4.53
**Right hemisphere: HM (n = 80) vs. HC (n = 57)**					
Inferior temporal gyrus (ITG)	646	47	− 71	− 25	− 5.98
Precuneus (PCUN)	249	7	− 76	50	− 5.24
Primary visual area 1 (V1)	361	21	− 102	− 7	− 5.02
Superior temporal gyrus (STG)	159	59	− 12	− 5	− 4.80
Superior parietal lobule (SPL)	129	29	− 79	46	− 4.74
Occipital Pole	153	21	− 101	15	− 4.69
Primary motor cortex (M1)	92	44	− 30	46	− 4.62

*HM* high myopic control, *HC* healthy control, *MNI* montreal neurological institute.

### Relationship between regional cortical thickness and clinical index

In the HM group, cortical thickness and ALM, the average macular thickness (AMT), KER1, KER2,the mean KER, the mean MFK, the refractive diopter were negatively correlated with the right orbitofrontal cortex (OFC), right dorsolateral prefrontal cortex (DLPFC) and right subcallosal cortex (P < 0.01, FWE-corrected;). We have also presented a flexible statistical result, as shown in Supplementary Figure S1–S7 (Please refer to supplementary materials).

**Figure 3:**
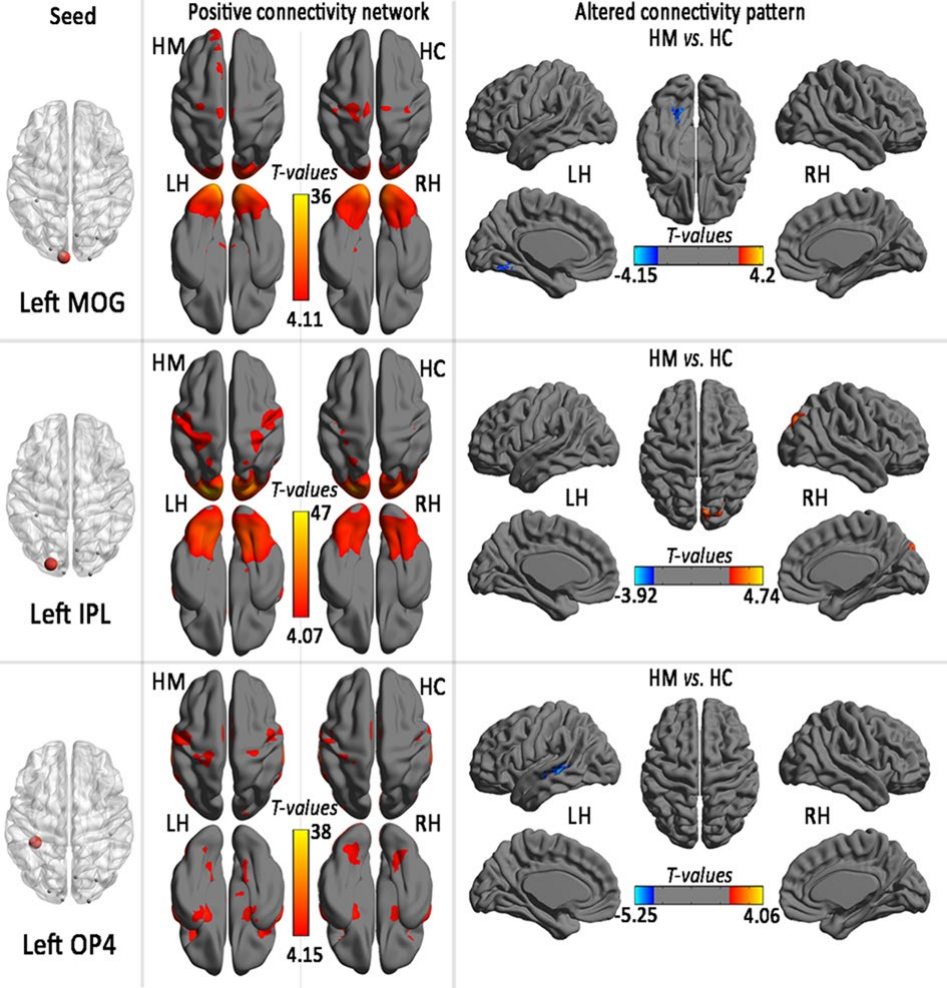
Positive connectivity network and group comparison in the region of interest in left hemisphere (seed) with altered regional cortical thickness in HM subjects (*LH* light hemisphere, *RH* right hemisphere, *HM* high myopic control, *HC* healthy control, *MOG* middle occipital gyrus, *IPL* inferior parietal lobule, *OP* parietal operculum).

**Figure 4:**
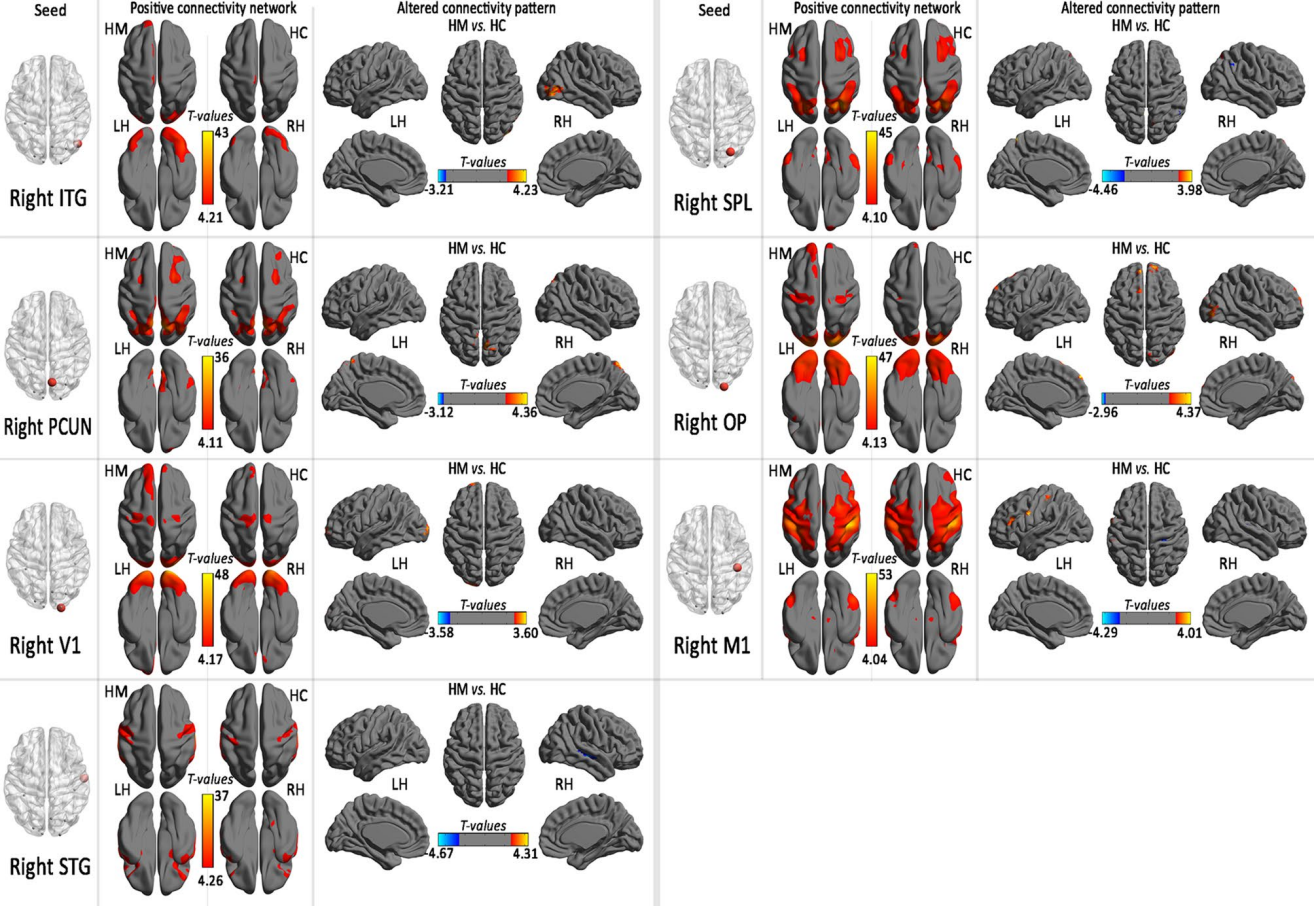
Positive connectivity network and group comparison in the region of interest in right hemisphere (seed) with altered regional cortical thickness in HM subjects (*LH* light hemisphere, *RH* right hemisphere, *HM* high myopic control, *HC* healthy control, *ITG* inferior temporal gyrus, *V1* primary visual area 1, *STG* right superior temporal gyrus, *SPL* right superior parietal lobule, *M1* right the primary motor cortex, *OP* parietal operculum, *PCUN* precuneus).

Within the positive connectivity regions, the HM subjects was detected significant thickness-related FC differences (shown in Table 3, Figs. 3 and 4).

**Table 3:** Regions showing thickness-related functional connectivity differences in the HM group.

Location	BA	Cluster size	MNI			T-values
			X (mm)	Y (mm)	Z (mm)	
**Left MOG (MNI: − 7, − 100, − 5; seed size: 10 mm)**						
Left Fusiform Gyrus	19	104	− 9	− 66	− 18	− 3.92
**Left IPL (MNI: − 21, − 91, 25; seed size: 10 mm)**						
Right Occipital_Sup/PCUN	19	135	21	− 84	33	4.14
**Left OP4 (MNI: − 36, − 39, 11; seed size: 10 mm)**						
Left STG/MTG	21, 22	158	− 45	− 36	0	− 5.21
**Right ITG (MNI: 47, − 71, − 25; seed size: 10 mm)**						
Right MOG	18, 19, 37	234	42	− 87	3	4.23
**Right PCUN (MNI: 7, − 76, 50; seed size: 10 mm)**						
Bilateral PCUN/right IPL	7	144	9	− 66	63	4.36
**Right V1(MNI: 21, − 102, − 7; seed size: 10 mm)**						
Left MOG	18, 19	51	− 24	− 102	6	3.60
Left SFG	10	40	− 12	63	0	3.48
**Right STG (MNI: 59, − 12, − 5; seed size: 10 mm)**						
Right STG/MTG	21, 22	96	51	− 21	− 3	− 4.16
**Right SPL (MNI: 29, − 79, 46; seed size: 10 mm)**						
Left IPL	40	24	− 48	− 51	39	− 3.56
Right IPL	40	48	42	− 51	33	− 3.66
Right SPL	7	22	24	− 69	57	3.26
Left SPL	7	29	− 6	− 57	66	3.91
**Right OP (MNI:21, − 101, 15; seed size: 10 mm)**						
Right MOG	18, 19	77	45	− 84	− 3	4.10
Right SFG	10	64	15	63	24	3.91
Right cuneus	19	45	15	− 93	33	3.76
Left SFG	9	52	− 6	57	39	4.42
Left SMA	6	25	− 6	27	66	3.70
**Right M1 (MNI:44, − 30,46; seed size: 10 mm)**						
Right STG	41, 13	21	48	− 36	15	− 4.26
Left IFG	46	62	− 42	30	24	3.45
Left Frontal_Oper	−	26	− 39	12	12	3.41
Left IFG	6	36	− 60	3	33	4.00
Left postcentral gyrus	2, 1	30	− 54	− 27	54	3.69
Right precentral/postcentral gyrus	3, 4	41	27	− 33	57	− 3.28

*BA* brodmann area, *MNI* montreal neurological institute, *MOG* middle occipital gyrus, *IPL* inferior parietal lobule, *ITG* inferior temporal gyrus, *V1* primary visual area 1, *STG* right superior temporal gyrus, *SPL* right superior parietal lobule, *M1* right the primary motor cortex, *OP* parietal operculum, *OFC* orbitofrontal cortex, *MTG* middle temporal gyrus, *SFG* superior frontal gyrus, *IFG* inferior frontal gyrus, *PCUN* Precuneus, *SMA* supplementary motor area.

## Discussion

In this study, we characterized the cortical thickness reduction and thickness-related FC differences in the HM group. According to the above results, in the HM, significantly thinning cortical thickness involving visual center and visual information processing area, but thicker cortical thickness only in multimodal integration region (left OP4). Furthermore, in the thickness-related FC, decreased FC was between left MOG and left fusiform gyrus, FC also decreased between the left ITG group and the right MOG group, but increased FC was between left IPL and right superior occipital/PCUN. Besides, ophthalmic clinical indicators were found negatively correlated with cortical thickness in several brain regions (right OFC, right DLPFC and right subcallosal cortex).

### Cortical thickness reduction in visual center and visual processing area

At first, we found that the HM affected regional cortical thickness in right visual center. Right V1 (sometimes called Brodmann's 17, BA17) is the first stage of cortical processing of visual information. Area V1 contains a complete map of the visual field covered by the eyes,V1 creates this map to guide the line of sight to the most prominent position. It has the function of visual data compression and data selection, which means that the attention is not focused, and the visual information will be difficult to be transmitted from V1 to the following channels V2, V4, etc.^18^. Thus, damage to V1 would lead to a loss of visual perception. Right occipital pole Right occipital pole corresponds to and controls the visual cortex (V1/BA17) and is the center of early visual processing, which is also responsible for speech processing and spelling to sound conversion^19^. Accordingly, it is likely that the damage to right occipital pole could contribute to speech process and visual processing, especially for visual anomalies, and likely to affect the V1 brain regions associated with the visual pathway. By the way, we found that both the right V1 and right occipital pole were presented as the thickness of cortex decreased from HM patients, so we speculated that maybe the interaction between these two areas of the brain associated with vision induces in HM patients.

Congruency-related processing was localized to the right superior occipital gyrus, which is part from the occipital cortex, once there has been abnormality on this brain area, the occurrence of eye-related diseases may be induced. Another separate study found that the FC of right calcining and right lingual gyrus was reduced with superior occipital gyrus in patients with genesis of the corpus callous (AgCC), such patients often have visual and sensorimotor abnormalities, they believe that functional reorganization may occur to visual-related brain regions, such as right superior occipital gyrus, to compensate for the abnormality of visual processing^20^.Furthermore, Left IPL is a subregion of the inferior parietal cortex, the latter is closely related to these functions of perception, motion orientation, context memory retrieval, language understanding, digital processing and social cognition^21^, besides, IPL area shows activation enhancements in syntax processing^22^. An MRI study about heroin addiction shows that the abnormal brain function of heroin addicts is related to ILP, the FC between left and right IPL brain regions of these untreated patients decreased significantly, these show that long-term dependence on heroin may will destroy the structure and function of IPL, besides, IPL may be the neural target for the intervention and treatment of this disease^23^. In addition, Shi L et al., compared and analysis the cortical thickness of patients with type 2 diabetes mellitus (T2DM) and T2DM complicated with hypertension at the same time, they found that patients with both diseases had thinner cortical thickness of IPL, thus, they believe that T2DM will cause cognitive impairment, and hypertension may accelerate the decrease of cortical thickness^24^. The above research reflects that long-term experience of a disease may lead to structural and functional changes of related brain areas, which may become the target areas for the treatment of the disease. According what we found that the cortical thickness of left IPL decreased and the FC between right superior occipital gyrus/PCUN and left IPL was increased in patients with HM. Thus, we speculate that these two brain regions may play a role in visual changes or be affected by long-term abnormal visual experience.

Left MOG is located in the left hemisphere, which participates in language, concept, number, analysis, logical reasoning and other functions, indeed, occipital gyrus is related to visual processing, especially early visual processing. Chen et al. found that the brain activation related to visual perception was mainly concentrated on the occipital and parietal cortex, while the early processing of visual awareness activation was located in bilateral posterior central gyrus and left MOG^25^. In addition, MOG participates in the perception of softness^26^, and it's more responsive to the texture than the location of the point^27^. Furthermore, MOG may retrieve visual information associated with the object being touched^28^. Moreover, left fusiform gyrus is located on the surface of temporal and occipital lobes, which is crucial to language function and is modulated by literacy^29^, what is more, it is also the key brain area for bilingual language processing^30^. Additionally, it has also been reported that the fusiform gyrus has a unique visual processing mechanism for text and objects^31^. Practically speaking, the left MOG and left fusiform gyrus were found to be co-related in cognitive, linguistic functions and visual processing^32^. Thus, combined with our findings of FC changes in these two brain regions in HM group, we speculate that left MOG and left fusiform gyrus may be associated with some human diseases, behaviors and so on at the same time. In view of this, on the one hand, we speculate that decreased FC between these two brain regions may be related to the pathological physiology of HM. Also, according to the result that regional cortical thickness of MOG in HM group was lower than HC group, we suspect that abnormal visual experience may cause structural changes in the MOG and even affect its function to some extent. Maybe, there will be differences between HM's tactile or the rest of the functions compare to normal people.

Right ITG is part of the visual pathway system^33^, also, creativity is one of the main functions of this area, Cameron Arkin et al. found that creativity was negatively correlated with the volume of the right ITG^33^. Meanwhile, the brain region mainly involves in learning, memory formation and object categorization. Indeed, the cortical surface thickness of right ITG in patients with HM was thinner, we speculate that HM may cause changes in the structure of the brain, and patients with HM may be creative abnormality. Otherwise, on the basis of preceding complaint that both the ITG and MOG regions related to the visual system, we also noticed that the decreased FC between left ITG and right MOG in HM group. However, the ITG is responsible for late visual processing, and the MOG is responsible for early visual processing. Besides, Zhang et, al used resting-state electroencephalography (EEG) found that sleep deprivation would lead to the decreased FC between left ITG and right MOG^34^ and lead to significant declines in cognitive function too, including attention, working memory and decision-making^35^, these may be related to the decrease of sensory gat. As for patients with HM, perhaps the occurrence of the disease will affect the normal function of the brain, leading to a decline in FC between left ITG and right MOG at the same time.

There are several other brain regions (right precuneus, STG, right SPL, right M1) in the HM group showed thinning of cortical layer thickness. Although they were not the direct control brain regions of vision, they showed this change, which may mean they were associated with vision of a way.

Right precuneus located in medial parietal lobe, which is one of the most crucial brain area of default mode network and receives inputs from various vestibular and multisensory cortical areas, such as the parietal sulcus, inferior parietal lobe and parietal cover^36^. Meanwhile, it is considered to be related to cognitive and executive functions such as visual processing, auditory processing, memory, motivation, emotional processing^37,38^. As for the visual processing, visual imagery and episodic　memory is the major^39^.STG is the surface of temporal neocortex, which contains two main parts: the Heschl's gyrus (HG) and planum temporal. Furthermore, right SPL is responsible for the execution of the action, which involved in formation of a network of mirror neuron system (MNS), the brain regions that activated when an individual performs an action or watches others perform similar actions^40^. Specifically, research shows the level of SPL activation may be age-related, a recent study have shown that the activity of SPL in adults is significantly larger than children^41^, another studies have found that children's motor observation and motor execution skills improve, which may be closely related to the corresponding nerve^42^, meanwhile, the changes of SPL brain structure in HM patients may be different in adults and children. Moreover, right MI is one of the principal brain areas involved in motor function, which locates in the frontal lobe of the brain, along a bump called the precentor gyrus. The role of M1 is to generate neural impulses that control the execution of movement, it is responsible for generating motion images and executing motion, however, simple movements, such as hand or joint movements, do not involve the brain region^43^. We noticed that in HM patients, the cortical thickness of the above four brain regions became thinner, combined with the functional characteristics of these brain regions, we hypothesized that abnormal visual experience may alter the structure of the four brain areas and may affect the corresponding cognitive, executive functions and other functions.

### Cortical thickness increase in left OP4

In this study, HM group showed that only left OP4 presented an increase in cortical thickness. The role of the OP4 is action-dependent processing of touch, studies have shown that stimulate fingers during exercise and rest, respectively, OP4 would show an increased responsiveness to tactile stimulation during movement^44^, this effect may be due to the enhancement of feedforward communication in the transmission of externally generated somatosensory information during self-generated motion^45^. To this end, we speculated that OP4 structural abnormalities may be associated with HM disease experience in a long time, and the tactile function of HM patient may be different from normal people.

### Relationship between cortical thickness and ophthalmic clinical indicators

In the end, in addition to find that HM may cause dysfunction of some brain regions, we also noticed that the values of regional cortical thickness of right OFC, right DLPFC and right subcallosal cortex were negatively correlated with many of clinical variables (ALM,AMT, KER1, KER2,the mean KER,the mean MFK, the refractive diopter). In recent studies Han-Dong Dan et, al in a regional homogeneity (ReHo) and FC study of retinitis pigmentosa (RP)^46^ found that bilateral bilateral lingual gyrus/cerebellum posterior lobe (LGG/CPL)-left IPLshowed negative correlations with best corrected visual acuity (BCVA) in RP individuals; moreover, the ReHo values of bilateral LGG/CPL were negative correlations between the duration of RP patients, they evaluated brain activity mainly through FC and ReHo, and revealed that the corresponding brain activity of RP patients would change. In another study, Huang et al.^47^ found that the bilateral bilateral lingual gyrus (LIGG)/ (cerebellum anterior lobe)CAL were correlated positively with the BCVA in PR patients, they suggest that the BCVA-related primary motors and visual-motor coordination areas of the brain are impaired in RP patients. Accordingly, combined with our findings, we surmised the changes in cortical thickness may be associated with some structural changes in the eyes in HM individuals. Furthermore, we speculated that the dysfunction of OFC, right DLPFC and right subcallosal cortex which might be closely related to HM.

However, there were several shortcomings in our present research. Firstly, our sample size is limited. Secondly, we didn't synchronously monitor the changes of retinal RNFL thickness and cerebral cortex thickness. Finally, we did not select patients with monocular HM as control group to enhance the correlation between HM and cerebral cortex thickness. In the future, we will focus on the specific mechanisms between HM and structural changes in brain regions, and try to expand the sample size and monitoring indicators, and improve the selection of control group, in order to provide meaningful guidance for the prevention and treatment of HM.

## Conclusion

In summary. Our results suggest that the thickness of multiple cortical layers in HM patients has altered, which suggests that HM may cause structural changes in the relevant brain regions, or the vulnerable regions further leading to the aggravation of myopia, or the above two mechanisms exist mutually. Our findings may provide a new breakthrough in exploring the neural mechanism of HM and provide a new direction for its treatment.

## Supplementary information

Supplementary file 1

## Data Availability

MRI data used to support the results of this study are available on request from the corresponding authors.
