# Supplementary material for: Evidence of cortical thickness reduction and disconnection in high myopia
Source: Sci Rep. 2020 Oct 1;10:16239. doi: 10.1038/s41598-020-73415-3 (PMC7530748; doi:10.1038/s41598-020-73415-3)
Supplement: Supplementary file 1 [file 41598_2020_73415_MOESM1_ESM.pdf]

Evidence of cortical thickness reduction and disconnection in high myopia

Ya-Jun WU[1], Na WU[1], Xin HUANG[1], Jie RAO[1], Li YAN[1], Ling SHI[1], Hui HUANG[1], Si-Yu LI[1], Fu-Qing ZHOU[2*], Xiao-Rong WU[1*]

1Department of Ophthalmology, the First Affiliated Hospital of Nanchang University, Nanchang 330006, Jiangxi Province, China

2 Department of Radiology, The First Affiliated Hospital of Nanchang University, Nanchang 330006, Jiangxi Province, China

Co-first authors: Ya-Jun WU and Na WU

Corresponding author: Xiao-Rong WU. Department of Ophthalmology, the First Affiliated Hospital of Nanchang University, Nanchang 330006, Jiangxi Province, China. wxr98021@126.com

Fu-Qing ZHOU. Department of Ophthalmology, the First Affiliated Hospital of Nanchang University, Nanchang 330006, Jiangxi Province, China. fq.chou@yahoo.com

This is a statistical result chart of the relationship between local cortical thickness and clinical indicators. The results showed: HM group cortical thickness of right orbitofrontal cortex (OFC), right dorsal lateral prefrontal cortex (DLPFC), and right mandibular cortex were negatively correlated with ALM, average macular thickness (AMT), KER1, KER2, average KER, average MFK and refractive power (P <0.01, FWE corrected)

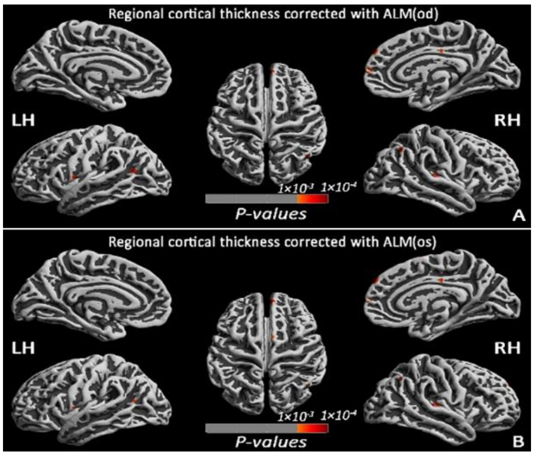

Figure S01. Regional cortical thickness was correlated with the ALM in the HM group (P < 0.001, without correction). (LH, light hemisphere; RH, right hemisphere; od, oculus dexter; os, oculus sinister; ALM, axis length)

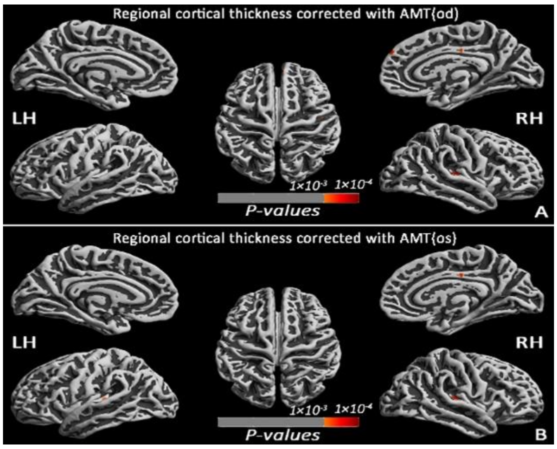

Figure S02. Regional cortical thickness was correlated with the AMT in the HM group (P < 0.001, without correction). (LH, light hemisphere; RH, right hemisphere; od, oculus dexter; os, oculus sinister; AMT, the average macular thickness)

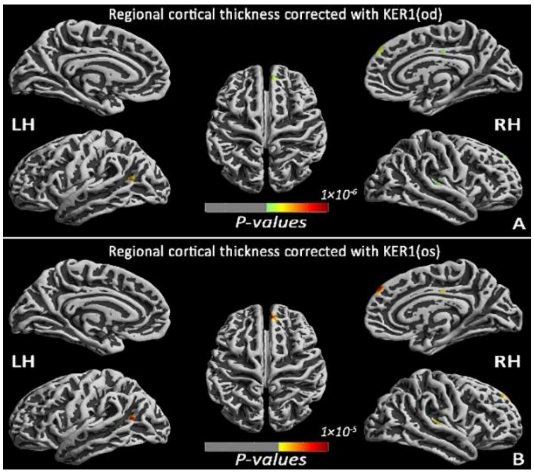

Figure S03. Regional cortical thickness was correlated with the KER1 in the HM group ($P < 0.001$, without correction). (LH, light hemisphere; RH, right hemisphere; od, oculus dexter; os, oculus sinister; KER, keratometer)

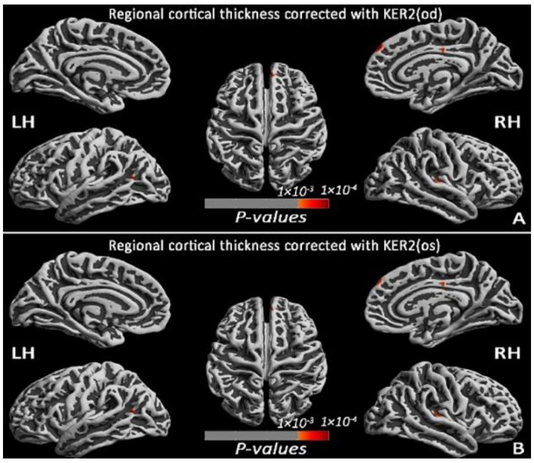

Figure S04. Regional cortical thickness was correlated with the KER2 in the HM group ($P < 0.001$, without correction). (LH, light hemisphere; RH, right hemisphere; od, oculus dexter; os, oculus sinister; KER, keratometer)

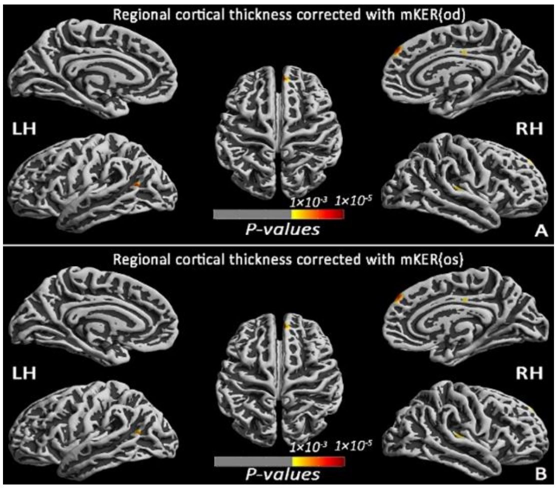

Figure S05. Regional cortical thickness was correlated with the mean KER in the HM group (P < 0.001, without correction). (LH, light hemisphere; RH, right hemisphere; od, oculus dexter; os, oculus sinister; mKER, the mean keratometer)

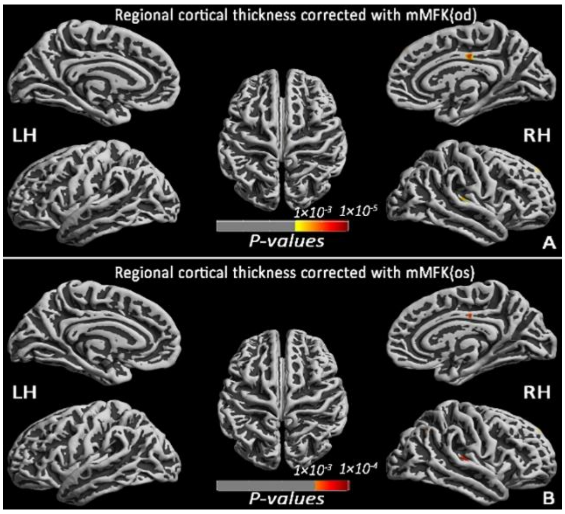

Figure S06. Regional cortical thickness was correlated with the mean MFK in the HM group (P < 0.001, without correction). (LH, light hemisphere; RH, right hemisphere; od, oculus dexter; os, oculus sinister; mMFK, the mean macular fovea thickness)

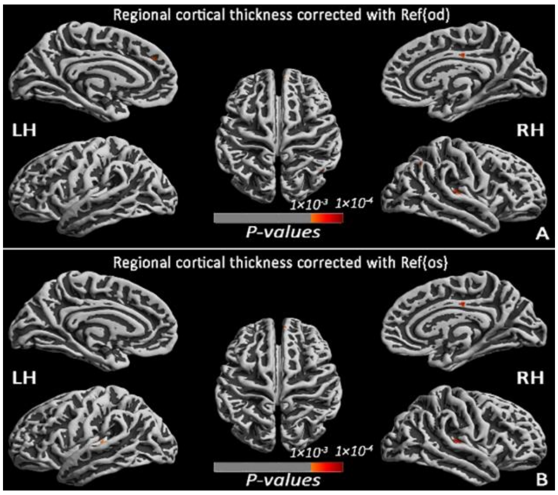

Figure S07. Regional cortical thickness was correlated with the refraction in the HM group (P < 0.001, without correction). (LH, light hemisphere; RH, right hemisphere; od, oculus dexter; os, oculus sinister; Ref, the refractive diopter)
